# “The Good, The Bad, and the Minimum Tolerable”: Exploring Expectations of Institutional Food

**DOI:** 10.3390/foods10040767

**Published:** 2021-04-03

**Authors:** Hanne Andreassen, Olga Gjerald, Kai Victor Hansen

**Affiliations:** The Norwegian School of Hotel Management, University of Stavanger, 4036 Stavanger, Norway; olga.gjerald@uis.no (O.G.); kai.v.hansen@uis.no (K.V.H.)

**Keywords:** food expectations, aging consumers, health services

## Abstract

There is a tendency towards greater expectations of consumer goods and services in society—what was once judged as ideal may now be a bare minimum. This presents a challenge for food providers in the upcoming decades. As the more demanding baby boomer cohort ages, health institutions of the future will face challenges meeting their food expectations. The purpose of this study was to explore expectation type dynamics and function with updated empirical material on aging consumers expectations of institutional food and advance our current understanding of how consumers evaluate their expectations. This qualitative study employed in-depth semi structured interviews with 14 informants between the age of 58–79. Content analysis was performed to capture the informants’ food expectations based on the expectation hierarchy proposed by Santos and Boote. Analyzing the content and relationship between different expectation types led to three main findings: expectation functions and content, interconnectedness, and the role of affect. Based on the findings, this study contributes by making several propositions for future research and proposes an updated expectancy–disconfirmation model. Importantly, this study provides novel knowledge that can help health institutions understand and meet aging consumers expectations of institutional food.

## 1. Introduction

Aging consumers are now recognized as more diverse compared to younger consumers with regards to needs, lifestyle and habits [1,2]. Particularly, as the baby boomer generation ages, these differences are becoming even more clear [3]. Baby boomers are known for being more demanding, having higher purchasing power, and being healthier than ever before, and they are accustomed to a more comfortable life than previous generations [3,4]. Moreover, they make up an increasingly large portion of society [5]. In other words, they are the next big generation to please. The aging population and a tendency towards greater expectations [6] creates especially large challenges for health services and institutions. How can health institutions prepare for the demands of the upcoming wave of elderly? Research shows that food in health institutions are important factors for the resident’s quality of life and well-being [7]. However, the current food situation in institutions is colored by institutional bias—consumers often hold negative attitudes towards the food served [8,9], and several studies highlight issues with the food in institutions [8,10,11]. The discrepancy between generally rising expectations and negative perceptions of institutional food is troublesome considering the increasing demand in the future [12]. In order to prepare for the future wave of elderly in health institutions, it is thus important to understand their expectations and how they are evaluated.

The present study focuses on aging consumers’ expectations towards food in health institutions, specifically hospitals and nursing homes, in a Norwegian context. Expectations is a multifaceted construct, and we lack updated empirical knowledge on expectations and their impact on satisfaction judgements today. In order to further our knowledge of pre-consumption expectations in current time, this paper seeks to empirically explore different types of expectations based on the expectation hierarchy proposed by Santos and Boote [13]. This framework is used as a basis for exploration, but not exhaustively. The context of institutional food is timely and important as the future consumers (baby boomers) are representing a shift towards greater expectations, and society face challenges in accommodating the upcoming wave of elderly. Therefore, the purpose of this paper is to explore expectation type dynamics and identify their functions in the context of institutional food and to incorporate these findings in into the expectancy-disconfirmation model [14].

Expectations are important to understand due to their role in consumer satisfaction [15,16]. The well-known expectancy-disconfirmation model [14] explains satisfaction as an additive function of expectations and perceptions, which results in disconfirmation. The theory holds that positive disconfirmation leads to satisfaction, and negative disconfirmation leads to dissatisfaction. The expectancy-disconfirmation model has been used to assess satisfaction and the effect of disconfirmation in a variety of contexts, such as in airports [17], public services [15,18,19], and tourism [20]. Furthermore, the widely used Service Quality Model [21] also compares expectations of the perceived service to determine service quality. In other words, the importance of expectations is well-established, however, the optimal definition and nature of expectations is still not fully understood.

Expectations are pre-trial beliefs about a product or service and its performance at some future time [22,23], and may be considered with or without comparison to the actual level of performance [24,25,26]. Put differently, expectations are judgements of what consumers think will or should happen under particular circumstances [27]. A complex issue with the expectation construct is different expectation types [13]—what the consumers realistically expects may be very different from what they ideally expect. Research suggests that consumers understand and use several levels of expectations simultaneously [28,29,30]. However, researchers disagree revolving the number of expectation types, the dynamics and interaction between them, and which type is optimal for measuring satisfaction. Previous studies show that measuring different expectation types will yield different satisfaction outcomes for the same product or service [18,31,32]. Therefore, it is important to further our understanding of how the expectation types function, as it can have a huge impact on satisfaction results.

Previous studies have documented several distinct expectation types, with the most cited types being predictive, normative, ideal, and minimum tolerable expectations [28,30,32]. In the consumer satisfaction/dissatisfaction (CS/D) literature, predictive expectations dominate [32] while in the service quality (SQ) literature ideal and normative types are mostly used [33]. More recently, the CS/D and SQ literature has been increasingly reconciled [32]. Santos and Boote [13] proposed a hierarchy of expectation types in their theoretical model of consumer expectations, bridging the CS/D and SQ literature. The hierarchy includes standards based on 56 expectation definitions, and were summarized as ideal, normative, desired, predicted, minimum tolerable, intolerable, and worst imaginable (see Santos and Boote [13] for expectation hierarchy). Table 1 contains explanations of the proposed expectation types.

Although a stream of research in the 1980s and 1990s made important progress on understanding the expectation construct [29,33,38], many of the ideas and propositions made then were never adequately tested. Sweeney et al. [39] state that research on expectations has almost vanished in recent years, despite the evolving and dynamic nature of expectations demanding it. Therefore, it is important to develop a more solid understanding of expectations, especially considering the tendency towards greater expectations. Notwithstanding, the theoretical findings based on research from several decades ago may not hold up due to the change in consumers lifestyle [6]. This is especially relevant for the aging consumer population who represent a “new old” compared to previous elderly generations [4,5,40]. Additionally, market trends demonstrate that consumers are increasingly concerned with what they eat, in terms of for instance healthiness, appearance and sustainability [41,42,43,44,45,46]. These two trends present a challenge for institutional food providers and updating our knowledge on consumer expectations in this context is crucial.

## 2. Methods

The study adopted a qualitative research design to explore the expectation construct in the specific context of institutional food. An explorative method is used to gain a deeper understanding of the construct, clarify concepts, and eventually propose hypotheses [47]. This was deemed an appropriate method for this study as we seek to revisit how consumers describe expectations and explore dynamics between the expectation types as an evaluation occurs. To do that, it is important to assign content and meaning to the expectation types and create a solid understanding of the contextual situation. In other words, a qualitative research design allows us to explore the expectations and the interplay between them and make propositions for further research. Fourteen in-depth semi-structured interviews were conducted and analysed using a content analysis approach [48]. Saturation was achieved after 13 interviews, and the last interview confirmed saturation. The Norwegian Centre for Research Data [49] approved the data collection (NSD reference number: 493572).

By agreement, the interviews were audio-recorded and subsequently transcribed for analysis purposes. All interviews started with an initial phase during which the participants were informed about the aim and nature of the study, how the data will be stored and how the participants can withdraw from the study (informed consent).

The interviewees were consumers between 58 and 79 years old, with a different experience of institutional food—ranging from direct, indirect (through close family members), and no experience. All informants were recruited in Norway. It was important to strive for variety among the participants in terms of age, experience, type of institution, and gender to broadly sample the domain of the expectations construct. Before recruitment, the inclusion and exclusion criteria for the study were established. An inclusion criterion was that the maximum age of the informant was 80 years of age and the minimum age was 55 years old. Based on statistics from SSB (2018) on institutionalized individuals and duration spent in institutions [50], we chose to use 80 years old as a mean estimate for elderly institutionalization, thereby the upper age limit. The minimum age was chosen based on population prognosis [51] and to include the baby boomer cohort (aged 55–75 in 2019), which is predicted to be increasingly different from previous generations of elderly [3]. Exclusion criteria were mature consumers outside the age range and people in the age group 55–80 currently admitted to institutions full-time (e.g., nursing homes) or part-time (rehabilitation centers). Table 2 provides descriptive information about the sample.

The informants were recruited using a purposive sampling technique. Before the day of the interview, the informants were sent an informational letter about the project. The informants were told that the institutions of interest were nursing homes and hospitals. All the interviews were held in places that were convenient for the informants, primarily in the informant’s homes. The interviews were conducted from June 2019 to September 2019. In-depth semi-structured interviews were used to explore the consumer’s expectations of the institutional food. This method allowed informants to provide detailed information that reflected their perspectives and gave the researcher the flexibility to address emerging areas of interest [52]. The interviews lasted approximately 45 min to 1 h.

The interview guide included questions about the participants’ expectations of the institutional food, perceptions, food habits, food-related personality traits, food preferences, and knowledge. Demographic questions were also included. In addition, we used established scales to formulate questions about food-related personality traits: food involvement (FIS) [53], food neophobia (FNS) [54], and health consciousness (HC) [55]. We have used 4 items from the FIS scale from the preparation and eating subscale. From the FNS scale, we used 2 items from the food neophobia subscale and 2 from food neophilia subscale. Three items from the HC scale were used: 1 from health consciousness subscale, 1 from health alertness subscale and 1 from health involvement subscale (see Table S1 in Supplementary Materials for list of items used). Notably, we did not measure the personality traits, but used the scale items as basis for a discussion about food personality. The questions about the expectation types were framed to capture the expectation type differences (see Table S2 in Supplementary Materials). Some of the expectation types were addressed directly, while others became clear indirectly through other topics. Progressively, the interview delved into the additional aspects revolving the institutional food experience. All interviews were recorded with the informant’s permission and transcribed for analysis.

The data was analysed in NVivo 12 software using a content analysis approach. Content analysis allows for flexibility in using a combination of inductive and deductive approaches [56]. The interviews were analysed and coded simultaneously as the data collection continued, and the interpretation of the data evolved over time. Codes were adjusted as new conceptions appeared and connections between the codes were established during the analysis. Transcription was completed by the first author who also developed an initial codebook. All three authors met weekly to discuss the core codes and the sorting of the codes into concepts and categories. When all three authors agreed on the main categories reached, quotes representing the categories were added to illustrate the relationships between categories, concepts, codes, and citations.

## 3. Findings and Discussion

Analyzing the content and relationship between different expectation types led to three main findings: expectation functions and content, interconnectedness, and the role of affect. The following sections presents each finding and makes propositions for future research and suggests to incorporate an extended expectation construct into the expectancy-disconfirmation model.

### 3.1. Expectation Functions

**Figure 1:**
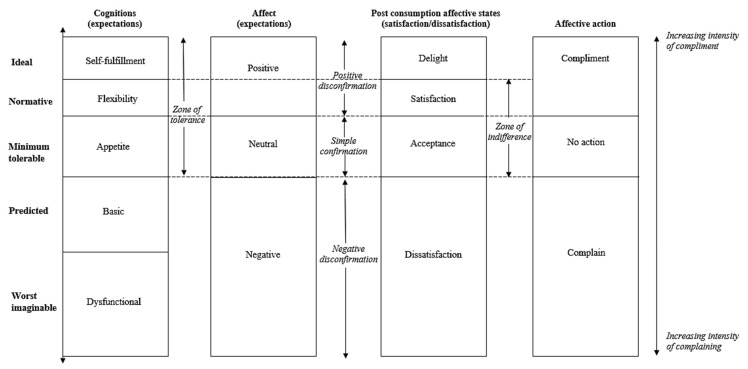
Extended Expectation Hierarchy.

From the empirical material, we were able to elicit several expectation types in the institutional food context: ideal, normative, deserved, predictive, minimum tolerable, and worst imaginable. Hence, this study supports the notion that consumers hold several expectation types simultaneously [32]. Through the analysis, we observed that the different expectation types were linked to specific consumption goals related to the institutional food experience. The focus of the content in the expectation types evolve from utilitarian (in the lower ranked expectation types) to hedonic consumption (in the higher ranked expectation types). In other words, in the lower expectations informants were concerned about quantity and practicalities revolving the food, while in the higher expectations hedonic values were more prominent. Moreover, our findings indicate three main functions of the expectation types: positive (the good), negative (the bad) and neutral (the minimum tolerable). Figure 1 illustrates our findings by providing a modified and extended expectation hierarchy based on the theoretical model by Santos and Boote [13]. The following sections presents and discusses the proposed functions, relative rankings, and consumption goals associated with each expectation type for the specific institutional food context.

**“The good”**

The positive expectations group consist of the types ranked above minimum tolerable, which in our study are the ideal, normative, and deserved expectation types (Figure 1). These are characterized by high hedonic focus and assert a positive influence on the total expectation’s evaluation and contribute to rise the expectations. the positive expectation types build on each other and the neutral expectations. Hence, although the presentation of the positive expectations does not address expectations directly related to food quality, presentation and eating environment, it does not mean that they are not present in the positive expectation types. In this paper, we have chosen to focus on what distinguishes the expectation types from each other and what is *not* present in the lower ranked type. Thus, in the positive expectations, we address what *more* they expect at the normative and ideal level *given* that the neutral expectations are met.

The highest level of expectations are the ideal expectations, which we suggest are linked to a "self-fulfilling food consumption". By this, we mean that the consumers ideally expect to be empowered to realize themselves and their preferences through their food consumption and choices. Importantly, most of the informants expect a high degree of freedom of choice and autonomy in the institutional food experience, as demonstrated by the following citation:

“The most important thing to me is freedom of choice… and a good glass of wine”

(male, aged 64)

To achieve a higher sense of autonomy, several of the informant’s mention having flexible menus as an important expectation, as illustrated in the citation below. To be able to choose where to eat, when to eat and with whom to eat were also mentioned as aspects of a self-fulfilling food consumption.

“What would be ideal, and a bit of a luxury, would be that when lunch and dinner time approaches, you would get a menu”

(female, aged 60)

Although scarcely researched in this context, previous studies have found choice and autonomy in the institutional food experience to be related to enhanced motivation to consume meals, food satisfaction and well-being [57,58,59]. Most of the informants in this study (particularly the younger part of the sample) stressed the importance of expectations related to freedom of choice and were aware that it is not the norm in nursing homes today. The informants often draw lines to other food situations, for instance restaurants, in describing their ideal expectations, these expectations appear to function as an ideal ceiling for the institutional food experience. We suggest that the ideal expectations assert a strong positive influence on the total expectation evaluation and may create unrealistically high expectations (considering current practices) due to using food experiences outside the institutional context as a reference point.

The detected normative (“should”) expectation type is explained as what the consumers think they should be able to expect. In our material, the normative expectations were more colored by the context than the ideal expectations, however still included untraditional aspects from the institutional perspective. This expectation type represents consumption goals related to what we define as "flexible food consumption". The informants discuss expectations of aspects related to food variety and various food types, often including reference to introducing more novel and international foods in institutions, such as Thai food, sushi, and tacos, as illustrated in the citation below. This appears to be especially important to the younger informants of the sample. Several of them stated that they gradually added new dishes to their diet and stayed updated on current “food trends”, for instance by incorporating more vegetarian options in their diet, ecological food, and traditional dishes from other countries.

“I definitely believe future residents will think they should be able to expect more international food in institutions. Today, we have a lot of variation in food because we have changed our diet to something completely different. Our diet is much more internationalized, and thus people will demand more choice alternatives. Before it was fish, potato dumplings and minced meat. Today, you need to add tacos and stuff like that”

(Male, aged 59)

Moreover, the expectation of flexibility in food consumption entails the possibility to have personalization and tailoring of the meals related to personal needs. Several informants point to the importance of having a diet that is nutritionally optimized to the specific health needs of each individual. A male informant underlines this in relation to the use of in-house or remote kitchen solutions for institutional food.

“The kitchen has to be placed in the institution if you want to serve local food and personalize the meals after needs, which is something I think should be prioritized”

(Male, aged 77)

We suggest that the normative expectations influence the other expectation types positively and provides a more realistic point of reference that still would be able to generate satisfaction (compared to ideal expectations).

The deserved expectations type appears to be linked to goals of "dignified food consumption". All the informants expressed that the elderly in institutions deserved the best, as illustrated in the citation below. This strong sense of equity for the elderly appear to function as an amplifier on the positive and neutral expectations and triggers affective responses when evaluating the negatively loaded expectations. It appears to set the tone for further evaluation of what one expects. Therefore, it could be that deserved expectations may be where the initial (or one of the initial) expectation formations occur before the consumers proceed to formulate their other expectation types. We suggest that the function of the deserved expectations constitutes an overall positive effect and contributes to raise the expectations.

“When you get in the last stage of life, then you should be able to choose what you want to eat yourself. It should not even be a question—the elderly should be satisfied and prioritized.”

(Female, aged 68)

**“The bad”**

The negative expectation group consist of the types below minimum tolerable: the predictive and worst imaginable types (Figure 1). The predictive (“will”) expectations in the material are predominantly negative, and they appear to function as a “damper” on the positive expectation types. The predictive expectations were clearly related to the informant’s perceptions of the institutional food and constitute a "basic food consumption". In this study, a basic food consumption is explained as having acceptable food quality and supply at a utilitarian level. The informants predicted the basic aspects of a meal would be fulfilled, however they think they will be faced with several constraints hindering an optimal meal experience, such as lack of staff, finances, social interactions, and restricted autonomy. The dampening function of predictive expectations and expectations of constraints is illustrated in the citations below.

“I would like to have varied food (as in internationalization). But you cannot really expect that I think… but ideally, I would like some variation”

(male, aged 70)

“I think there are miles of distance between the reality of institutional food, and how it should be”.

(Female, aged 73)

Contrary to previous research and the proposed model by Santos and Boote (2003), this study suggests that the predictive expectations are negative. This could be due to the context and institutional bias [8,9]. However, this could also be the case for other consumption contexts where the anticipated outcome is potentially negative, especially health services may be susceptible to this phenomenon. Further research is needed to determine whether this is a contextual matter, or a matter of generally higher expectations in society that are harder to meet.

The worst imaginable expectation type entail expectations of a "dysfunctional food consumption". Specifically, it includes expectations of insufficient food quality, quantity, and appetite-activation. These expectations induce fear and hopelessness in the informants, who were clearly affected when discussing this topic, for instance by expressing anger or frustration. These expectations appear to be based predominantly on media stories and word of mouth, as demonstrated in the citation below. Evidently, these expectations assert a strong negative influence on the other types and the expectations in total. In particular, the predictive expectations are often discussed in relation to the worst imaginable expectations.

“My neighbor had her husband in a nursing home. He was so thin because he was sick, but let me tell you, he lost even more weight up there (at the nursing home), and he was not the only one. Everyone talked about it, that they lost weight there due to insufficient food”

(Female, aged 78)

**“The minimum tolerable”**

The neutral expectations consist of the minimum tolerable expectation type which we found to act as a baseline for what is acceptable. In our material, this baseline appears to be set relatively high compared to perceptions. We found this expectation type to be related to an "appetite inducing food consumption", meaning that the food and eating environment is set to stimulate appetite in the consumers. This expectation type contains a certain level of hedonic aspects in addition to the utilitarian focus. The informant’s express expectations of social eating, for the food to have good taste and quality, to be nutritious and provide a sensory appeal. In other words, these informants at a minimum level expect the food and food experience to induce appetite in sensory and social regards.

“The food should be healthy and taste good. And adequate portions. That’s really at a minimum level. If I were to go a bit above that, I would say it should look appetizing—but that is also minimum actually”

(female, aged 60)

Interestingly, the empirical material indicates that minimum tolerable expectations are higher than predictive expectations. This is evident in our data as the predictive expectations are largely negative or modest at best, while the minimum tolerable expectations are positive and related to both sensory and social aspects of the food experience. The citations below illustrate the difference between the predictive and the minimum tolerable expectations, respectively.

Predictive: “Realistically, I’m not expecting any restaurant luxury. But that it is an average Norwegian diet”

(male, aged 64)

Minimum tolerable: “It should be good warm and appetizing food. In a cozy environment. That is at a minimum level. And good service. It’s important for the atmosphere”

(Female, aged 60)

Based on the model by Santos and Boote [13] switching the placing of these expectation types maps out a new zone of tolerance (Figure 1). The zone of tolerance (ZOT) is defined as ranging from ideal to minimum tolerable expectations and is explained as the range of service a consumer is willing to accept [33]. Therefore, this change in placement will have implications suggesting that consumers are harder to please than first assumed, as the zone of tolerance becomes narrower in this case. Moreover, it challenges some of the basic assumptions of ZOT theory [33]. As evident from the extended model based on our results (Figure 1), the zone of tolerance is narrower and subsequently impact the zone of indifference. Importantly, having higher minimum tolerable expectations than predictive expectations leads to a much higher risk of negative disconfirmation and thereby complaint behaviors, as the area of acceptance is smaller (see Santos and Boote [13] for original model and area of acceptance). Our extended model illustrate how it will be relatively more difficult to meet the expectations at the acceptable side of the line.

Contrary to previous research [13,33], our findings indicate that minimum tolerable expectations may be higher than predictive expectations. One exception is a study by Dean [31], who suggests that adequate expectations (similar to minimum tolerable expectations) are different from and relatively higher than predictive expectations in call centers. Dean [31] highlighted the importance of more research to establish the position of this expectation type. The present study contributes to do this by suggesting minimum tolerable expectations’ relative ranking on an established scale of expectation types based on empirical data.

### 3.2. Interconnected Expectations

This study indicates that consumers hold several expectation types simultaneously and that they range on a spectrum, similar to the expectation hierarchy proposed by Santos and Boote [13]. The expectation types appear to be working dynamically together and influence each other as the consumers discuss their expectations of institutional food. We observe this tendency to varying degree among all the detected expectation types, whereas some of the types increase or decrease the level of other expectations. For example, the predictive expectations may have a dampening effect on the ideal expectations in the institutional food context.

“Ideally, I would expect to be able to choose whatever I want (to eat), but realistically I guess you cannot expect that”

(Female, aged 73)

Another example of this is that the deserved expectations could influence and raise the minimum tolerable expectations, as demonstrated in the following citation:

“When it comes to elderly in nursing homes, I think we should not talk about a minimum. They have paid their taxes for all their years, so that is no way to treat the elderly. Yes, perhaps they don’t need a 5-course dinner and wine every single day. But it should be good quality, healthy food, presented nicely, in a social, home-like environment. That would be a minimum requirement”

(Female, aged 60)

Expectations may be more complex than assumed due to the continuous interplay in how consumers evaluate their expectations. The norm among scholars in measuring expectations in the expectancy-disconfirmation paradigm is choosing one or a few expectation types and measuring them independently. For instance, in CS/D literature, predictive expectations are most frequently used. The consequence of choosing a suboptimal expectation type to measure can have implications for the anticipated satisfaction results by providing an imprecise foundation of judgment. For instance, if one were to measure only the predictive expectations in relation to perceptions in the institutional food context, one would likely get an overrated satisfaction level due to the low predictive expectations. Considering the findings from this study, we have indications that meeting the predictive expectations of institutional food only would not necessarily generate satisfaction. Put differently, measuring for instance ideal and predictive expectations separately in an expectancy-disconfirmation model would yield very different and potentially inaccurate satisfaction results. By conceptualizing and capturing multiple expectation types, this could be avoided.

Based on our analysis, we make the following propositions for future research:

P1: The expectation types are interconnected and have the potential to influence each other.

P2: Consumers hold several expectation types simultaneously.

To further understand how expectation types influence each other, we suggest weighing the expectation types according to their function. Based on the observed content and function of the expectation types, we argue that the expectation types hold different weights that range from positive to negative that will impact the overall expectation judgment. For further testing, we propose weighting the specific expectation types according to their outcome importance and impact. In other words, how much will meeting the expectation types positively or negatively influence the rest of the model.

P3: The expectation types hold different weights (positive, negative, and neutral) that determines their function in relation to the other expectation types.

Further, we propose a modified hierarchy of expectations based on previous work by Santos and Boote [13], with changes in order of expectation types. Most important, the shift between the predictive and minimum tolerable expectation types could potentially create a narrower zone of tolerance which would make the institutional food consumers harder to please.

P4: Predictive expectations are lower than minimum tolerable expectations in the institutional food context.

Another avenue for future research is individual relative importance. From our material, we observe that some informants put more emphasis on certain expectation types than others. For instance, some informants appear to emphasize the negative expectations more than the positive expectations, and vice versa. Based on some tendencies in our qualitative material, we propose for further research to investigate how and if individual characteristics, such as food-related personality traits, influence the expectations the consumers hold in this context.

P5: Individual characteristics influence relative importance of expectation types in the institutional food context

### 3.3. Affective Expectations

**Figure 2:**
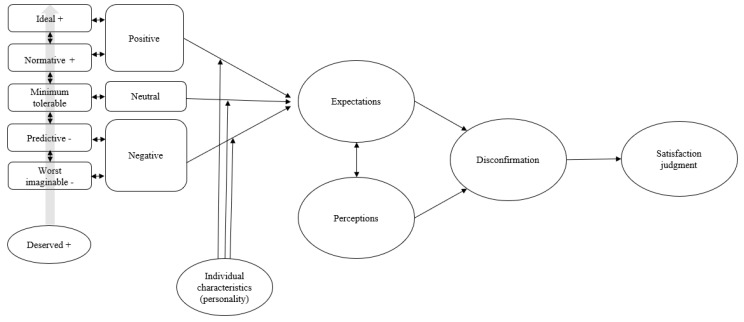
Proposed extension of the Expectancy-Disconfirmation Model.

While cognitive expectations consist of what consumers expect to happen, affective expectations address what the consumers expect to *feel like* in an upcoming experience [60]. Literature from psychology has researched this dimension of expectations, and the Affective Expectations Models holds that affective expectations shape the affective experience [61]. According to the AEM, affective expectations (i.e., how much you think you will enjoy a meal) are as important in determining affective reactions (i.e., how much you actually enjoy the meal) as the information gathered during the actual consumption experience (i.e., the relative quality of the meal). This notion has been supported in a variety of research [60,62]. In other words, how you expect to feel, not just what you expect to happen, could influence how you judge the actual consumption experience. The affective pre-consumption dimension of expectations is thus important to understand in relationship with the cognitive pre-consumption expectations. Based on the interviews, we assume a tendency for the cognitive expectations to trigger affective expectations. For instance, the affective expectation of food joy is mentioned in relation to the expectation of social eating, and discussing negative expectations triggered sadness, as illustrated in the citations below. Therefore, we suggest an added dimension to the model based on the accompanying affective expectations of cognitive expectations as illustrated in Figure 2.

“My mother did not want to go out of the room. She felt sad about the situation (in the institution). So, she stayed in her room and ate, and then she ate less and less. The food was halfway ruined when it came. It is so sad, horrifying, I think”

(Female, aged 60)

“It would bring a lot of joy to sit around a table with someone you care about and share a good meal with”

(Female, aged 68)

We propose that affective expectations should be further explored in relation to cognitive expectations. The empirical data indicate that affective expectations are present, and previous research demonstrates that it has important implications for the ultimate satisfaction judgements [61,62]. Therefore, we propose that cognitive expectations (what you expect to happen) is related to affective expectations (what you expect to feel like when something happens). This requires further testing.

P6: Affective expectations are related to cognitive expectations, and disconfirmation of affective expectations will influence the ultimate satisfaction judgement.

P7: Cognitive expectations (positive, neutral, negative) generate positive, neutral, and negative affective expectations.

### 3.4. Extended Expectancy-Disconfirmation Model

Based on our empirical findings and propositions, we suggest an extended expectancy-disconfirmation model to test quantitatively in the institutional food context (Figure 2).

### 3.5. Limitations

Among the limitations of this study is a restricted sample size and generalizability because of the context. However, the research design brought forward valuable insights and knowledge about the expectations construct and expectation evaluation that has not been explored in the institutional food setting before. The data was coded and analyzed by the first author. Despite the authors’ efforts to discuss the codes to avoid bias in interpreting the data and considering several plausible interpretations of the data, it is impossible to eliminate the possibility of bias. Further validation and exploration of the expectation types in institutional food should be undertaken through quantitative methods. The interview guide was tested repeatedly to remove unnecessary questions; however, it could have had limitations. It is also important to consider the potential issues with verbalizing expectations for an experience that is anticipated in the more distant future. The sample may have been narrow since all the informants lived in the same municipality in Norway. Nevertheless, the informants provided a broad representation of answers to the questions. Future studies should use larger samples to include multiple segments of elderly and further explore our findings quantitatively.

## 4. Conclusions

In the upcoming decades, understanding food expectations is important, especially for institutions that will meet the challenge of catering to the increasingly large and demanding aging population. This study implies that changes in consumer lifestyle and food habits are reflected in expectations towards institutional food. Self-fulfillment through food choices and flexibility as in offering international and tailored foods appear to be especially important expectations that should be prioritized in institutions such as nursing homes in the future.

Revisiting expectation theory and the expectancy disconfirmation paradigm is called for in these changing times. The present study contributes to the literature by providing updated insights on how consumers evaluate their expectations in the institutional food context. Our findings indicate that the expectation types may be highly interconnected, and we therefore suggest considering how they influence each other by giving them specific weights representable for their function on the total expectation evaluation. In other words, this study contributes to expand the meaning of expectations by incorporating several expectation types in the expectancy-disconfirmation paradigm. If possible, it would be of great practical use for researchers and marketers to provide a measurement instrument to capture multiple aspects of expectations and yield more accurate results in expectancy-disconfirmation models. Further, contrary to previous research [13] the findings indicate that minimum tolerable expectations may be higher than predictive expectations, which would have implications for ZOT theory compatible with the tendency towards greater expectations. In other words, the bar of what is considered acceptable could be raised and the consumers may be harder to please. This is interesting to explore further, especially with regards to aging consumers and baby boomers, change in consumers lifestyle and the continuously competitive marketplace. Lastly, we make several suggestions to further research on expectations by incorporating affective expectations with cognitive expectations and propose an extended expectancy-disconfirmation model for quantitative testing.

## Figures and Tables

**Table 1:** Expectation types definitions and characteristics.

Expectation Type	Definition	Characteristics
Ideal	The highest level of expectations Explained as i.e., the «wished for» level of performance [34].	Based on needs and wants [13]. Stable over time [35].
Should (normative)	What a customer feels a service should offer, rather than would offer [36].	Based on persuasion-based antecedents or the market supplier [23]. Stable over time [18].
Desired (want)	The level of performance that consumers want or hope to receive [13]	Based on mix of realistic predictions of what “can be” and what “should be” [13].
Predicted (will)	What a consumer expects predict will or is likely to happen in the next interaction with the service or product [25].	Based on past experience and perceived past performance. Less rigid than above types [18].
Minimum tolerable (adequate)	The minimum acceptable baseline of performance [34].	Implications for zone of tolerance—from ideal to minimum tolerable expectations: the extent to which consumers accept heterogeneity [33].
Intolerable	A level of performance or a set of expectations the consumer will not accept [37].	May stem from word of mouth, personal experiences, bad memories [13].
Worst imaginable	The worst imaginable scenario in a given context—the “worst case scenario” [13]	May stem from media (television, news, social media, radio) [13].
Deserved	The consumers view on the service encounter they fell they appropriately deserve [22].	Related to equity theory. Can interact with any of the other expectation types from normative to minimum tolerable [13].

**Table 2:** Sample demographics. Experience with institutional food: direct personal experience, indirect: experience of relatives, friends, etc.

Informant ID	Gender	Age	Marital Status	Experience with Institutional Food	Impression of Institutional Food
ID1	Male	77	Married	Direct	Negative
ID2	Female	68	Married	Indirect	Negative
ID3	Male	70	Married	Indirect	Positive
ID4	Female	58	Married	None	Neutral
ID5	Female	79	Single	Indirect	Negative
ID6	Female	78	Married	Indirect	Negative
ID7	Female	73	Single	Direct and indirect	Negative
ID8	Male	59	Single	Direct and indirect	Positive
ID9	Female	60	Married	Direct and indirect	Negative
ID10	Male	66	Single	None	Neutral
ID11	Male	64	Married	Indirect	Neutral
ID12	Female	60	Married	Indirect	Negative
ID13	Female	73	Single	Direct and indirect	Negative
ID14	Female	77	Married	Direct and indirect	Negative

## Data Availability

Not applicable.

## Supplementary Materials

The following are available online at https://www.mdpi.com/article/10.3390/foods10040767/s1, Table S1: Interview guide—food-personality; Table S2: Interview guide—capturing different expectation types.
