# Supplementary material for: “The Good, The Bad, and the Minimum Tolerable”: Exploring Expectations of Institutional Food
Source: Foods. 2021 Apr 3;10(4):767. doi: 10.3390/foods10040767 (PMC8066764; doi:10.3390/foods10040767)
Supplement: Supplementary file 1 [file foods-10-00767-s001.pdf]

Supplementary Materials

Table S1. Interview guide – food-personality

**Food-related personality trait**	**Items**
Food Involvement – Bell and Marshall (2003)	- I don't think much about food each day<br>- I enjoy cooking for myself and others<br>- Talking about what I ate or am going to eat is something I like to do<br>- Compared with other daily decisions, my food choices are not very important
Food neophobia - Pliner and Hobden (1992)	- I am constantly sampling new and different foods<br>- I like food from different countries<br>- I don't trust new foods<br>- I am afraid to eat things I have never had before
Health consciousness – Gould (1988)	- I am very self-conscious about my health<br>- I am alert to changes in my health<br>- I notice how I feel physically as I go through the day

Table S2. Interview guide – capturing different expectation types

**Expectation type**	**Interview guide**<br>**Expectation type basis**
Ideal	In an ideal world, what expectations do you have to the institutional food experience?<br>What do you think it ideally should be like?
Should/normative and desired	What do you think you should be able to expect of institutional food today?<br>What do you desire from the institutional food experience?
Predictive	What do you realistically expect from the institutional food experience?<br>What do you predict the institutional food will be like?
Minimum tolerable	What is the least you should be able to expect from institutional food experiences?<br>What are the minimum requirements for you to be satisfied with institutional food?
Worst imaginable and intolerable	Questions were not directly asked about these types; however, expectations of this type surfaced when informants discussed where they got the information t about institutional food, often from media stories and word of mouth.<br>If not addressed by the informants on their own initiative, the interviewer would ask: "What do you think is absolutely intolerable/ worst imaginable about the institutional food experience?".
Deserved	Questions were not directly asked about this type, but it appeared during the discussions in relation to the other expectation types.<br>If not addressed by the informants on their own initiative, the interviewer would ask: "What do you think the residents deserve from the institutional food experience?".
